# Transcriptomic and Physiological Variations of Three Arabidopsis Ecotypes in Response to Salt Stress

**DOI:** 10.1371/journal.pone.0069036

**Published:** 2013-07-23

**Authors:** Yanping Wang, Li Yang, Zhimin Zheng, Rebecca Grumet, Wayne Loescher, Jian-Kang Zhu, Pingfang Yang, Yuanlei Hu, Zhulong Chan

**Affiliations:** 1 Key Laboratory of Plant Germplasm Enhancement and Specialty Agriculture, Wuhan Botanical Garden, Chinese Academy of Sciences, Wuhan, Hubei Province, China; 2 University of Chinese Academy of Sciences, Beijing, China; 3 Shanghai Center for Plant Stress Biology and Institute of Plant Physiology and Ecology, Chinese Academy of Sciences, Shanghai, China; 4 Department of Horticulture, Michigan State University, East Lansing, Michigan, United States of America; 5 Department of Horticulture and Landscape Architecture, Purdue University, West Lafayette, Indiana, United States of America; 6 College of Life Sciences, Peking University, Beijing, China; University of Toronto, Canada

## Abstract

Salt stress is one of the major abiotic stresses in agriculture worldwide. Analysis of natural genetic variation in Arabidopsis is an effective approach to characterize candidate salt responsive genes. Differences in salt tolerance of three Arabidopsis ecotypes were compared in this study based on their responses to salt treatments at two developmental stages: seed germination and later growth. The Sha ecotype had higher germination rates, longer roots and less accumulation of superoxide radical and hydrogen peroxide than the Ler and Col ecotypes after short term salt treatment. With long term salt treatment, Sha exhibited higher survival rates and lower electrolyte leakage. Transcriptome analysis revealed that many genes involved in cell wall, photosynthesis, and redox were mainly down-regulated by salinity effects, while transposable element genes, microRNA and biotic stress related genes were significantly changed in comparisons of Sha vs. Ler and Sha vs. Col. Several pathways involved in tricarboxylic acid cycle, hormone metabolism and development, and the Gene Ontology terms involved in response to stress and defense response were enriched after salt treatment, and between Sha and other two ecotypes. Collectively, these results suggest that the Sha ecotype is preconditioned to withstand abiotic stress. Further studies about detailed gene function are needed. These comparative transcriptomic and analytical results also provide insight into the complexity of salt stress tolerance mechanisms.

## Introduction

Salinity is an increasingly important agricultural problem. Salt stress involves a combination of dehydration or osmotic-related stress effects and damage due to excess sodium ions [1] that greatly affect plant growth and crop production. Salt stress treatment also regulated the expression level of many genes involved either directly or indirectly in plant protection [2], [3]. Plant adaptations to salt stress include avoidance by reduced sodium uptake, sequestration of toxic sodium ions away from the cytoplasm, or production of compatible solutes or osmoprotectants to reduce molecular disruption [4], [5]. Much effort has been directed toward understanding the molecular mechanisms of plant salt tolerance, with the ultimate goal of improving salt tolerance of crop plants. Engineered salt stress resistance has been achieved by over-expression of genes encoding compatible solutes [6], ion transporters [7], and transcription factors [8] and is a high priority for commercial and public improvement efforts.

Besides genomics and mutant approaches, research based on the analysis of natural genetic variation in Arabidopsis and other species is receiving increased attention [9], [10]. Recently, large-scale evaluation of salt tolerance among different Arabidopsis ecotypes was performed by several groups [11], [12], [13]. Several loci associated with the salt sensitivity response were also mapped [14], [15]. Elemental profiling of shoot tissue from Arabidopsis ecotypes also revealed different Na^+^ and K^+^ accumulation because of natural variants of *AtHKT1* gene [16], [17], indicating possible natural variation of salinity tolerance in Arabidopsis [18]. Global transcriptome analyses have revealed numerous differences in transcript abundance among Arabidopsis ecotypes in response to several abiotic and biotic stresses [19], [20], [21]. Indeed, thousands of genes are differentially expressed between pairs of different Arabidopsis ecotypes under stress conditions [22], [23]. These differentially expressed genes were enriched for those involved in biotic and abiotic responses, suggesting that natural variation for gene expression is frequently observed among different Arabidopsis ecotypes. However, different ecotypes can differ for a large number of genes that are differentially regulated upon the same treatment [24], [25].

The Shahdara ecotype (Sha; also referred to as Shakdara) has been considered to be more tolerant to drought stress [26], osmotic stress [27], [28] as well as salt stress and ABA treatment [14], [15], [29]. These results are consistent with its origin in a region of overall low precipitation (the Shakdara valley of Tadjikistan; [30]). Quantitative genetics studies using different Arabidopsis ecotypes revealed a large variation for root development and seed germination under salt stress conditions. Twenty two quantitative trait loci (QTL) for these traits have been detected by phenotyping two recombinant inbred line populations, Sha × Col and Sha × Ler [14]. Another study indicated that a premature stop codon resulting in a truncated Response to ABA and Salt 1 (RAS1) protein in Sha contributes to the increased salt tolerance [15] based on QTL mapping.

To date, transcriptomic and physiological level changes between Sha and other ecotypes under salt stress conditions remain to be elucidated. To narrow down the list of candidate genes differentially expressed among Arabidopsis ecotypes under stress conditions, one salt tolerant (Sha) and two relative salt susceptible ecotypes (Landsberg *erecta* (Ler) and Columbia-0 (Col)) were used in this study to characterize transcriptome changes after salt treatment. The aims of this study are: (1) to detect physiological changes of three ecotypes under salt stress conditions; (2) to characterize transcriptional variation among these three ecotypes in the presence and absence of salt; and (3) to interpret related pathways which are involved in salt tolerance of the Sha ecotype.

## Materials and Methods

### Plant materials and growth conditions

Three *Arabidopsis thaliana* ecotypes Shakdara (Sha), Landsberg *erecta* (Ler) and Columbia-0 (Col) were used in this study. Arabidopsis seeds were surface sterilized and sown on Murashige and Skoog (MS) agar plates containing full-strength MS salts, 0.8% (w/v) agar, and 3% (w/v) sucrose. The seeds were stratified at 4°C for 4 days in darkness and then transferred to a growth chamber with 16 h/8 h light (350 μmol m^−2^s^−1^)/dark cycle at 23°C, or the seeds were directly sown in soil after stratification under the same conditions.

### Salt tolerance test

The standard for measuring germination rates was percent of seeds with emerged radicles (>1 mm) and/or two cotyledons turning green, as described by Wang *et al*. [31]. The root elongation data was quantified as described by Verslues *et al*. [32]. Salt treatment in soil-grown plants was initiated at 14 Days After Sowing (DAS) as described by Chan *et al*. [2]. Each ecotype-treatment combination included five replicate pots with nine plants per replicate pot. The NaCl concentrations were increased stepwise to the final concentrations (0, 100, 150, or 200 mM) by 50 mM every 2 days. Nine plants were grown in each pot and four replicated pots were used for each ecotype and salt treatment combination in above mentioned growth chamber. All the above experiments were repeated at least three times.

### Measurement of electrolyte leakage

Electrolyte leakage was determined from the detached aerial parts of salt-stressed plants with the indicated NaCl concentrations at the 10^th^ day after salt-treatment initiation. The detached plants (n = 10) were individually placed in 50 ml tubes containing 15 ml ddH_2_O and gently shaken for 2 h. Plants in tubes were then boiled at 100°C for 40 min. When plants were cooled to room temperature, the percentage of electrolyte leakage was determined as the percentage of the conductivity before and after boiling of the detached plants.

### Determination of reactive oxygen species (ROS) levels and enzyme activities

Superoxide radical and hydrogen peroxide (H_2_O_2_) were detected as described previously by NBT (Sigma-Aldrich) and DAB staining (Sigma-Aldrich), respectively [33]. Quantification of H_2_O_2_ content was determined using the method described by Hu *et* *al*. [34]. Two-week-old plants grown in soil were treated with 0, 100, 150 or 200 mM NaCl as described above. After 10 days, whole plants (0.2 g, FW) were ground in liquid nitrogen to extract total proteins and suspended in 500 µl PBS buffer (50 mM, pH 7.5), and then centrifuged at 12000 rpm at 4°C for 15 min. The supernatant was recovered and immediately used for enzyme activity measurement. Superoxide dismutase (SOD) activity was determined using the WST (2-(4-iodophenyl)-3-(4-nitrophenyl)-5-(2,4-disulfophenyl)-2H-tetrazolium, monosodium salt assay) method as described by Ukeda *et* *al.* [35]. One unit SOD was defined as the amount of enzyme required to inhibit reduction rate of WST-1 by 50%. The activity of catalase (CAT) was analyzed as described by Aebi [36]. One unit of CAT activity was defined as the amount of enzyme necessary to catalize 1 µmol H_2_O_2_ in 1 min at 25°C (pH 7.0). The absorbance of reaction buffer was analyzed at 520 nm. Experiments were repeated two times.

### Plant growth and salt treatment for microarray experiment

Seeds of three ecotypes were sown in MS plates as described above with three replicates for each ecotype and salt combination. Ten days after sowing, uniform seedlings of all ecotypes were carefully transferred to MS plates with or without 100 mM NaCl. Seedlings in MS plates without salt were used as controls. Plant materials were collected for RNA extraction 4 days after transferring at 2 h after dawn (the onset of illumination).

### RNA extraction and array hybridization

Total RNA was extracted and purified from leaves of at least 30 seedlings per plate for each ecotype and salt combination using QIAGEN-RNeasy Mini Kit (Qiagen, Valencia, CA, USA) according to guidelines specified by the manufacturer. Two biological replicates were prepared for each combination. Microarray analysis was performed using Agilent-021169 Arabidopsis 4 Oligo Microarray (V4) (Probe Name version). In total 150 ng of total RNA was used to prepare Cyanine-3 (Cy3) labeled probe with the help of the low RNA input linear amplification/labeling kit (Agilent technologies). Labeled cRNA probes (1.65 µg) were fragmented using fragmentation buffer (Agilent Technologies) and hybridized to the Arabidopsis arrays according to manufacturer’s instructions.

### Microarray data analysis

The arrays were scanned using the high resolution array scanner (Agilent technologies). Array images were acquired with Agilent's dual-laser microarray scanner and signal intensities were extracted from the scanned images with dedicated Agilent Feature Extraction software (Agilent technologies). GeneSpring software (Agilent technologies) was used to calculate the intensity ratios and fold changes. All the genes with a *P*-value below 0.01 and a fold change above 2 were chosen for further analysis. The normalized microarray data have been submitted to the Gene Expression Omnibus (GEO) database with accession number (GSE40940). Genes significantly changed by at least one comparison (*p*-value ≤0.05 and fold change >2.0) are listed in Table S1.

### Biological enrichment and metabolic pathway analysis

All genes with P-value <0.01 and fold change >2.0 were loaded and annotated in the Classification SuperViewer Tool (http://bar.utoronto.ca/ntools/cgi-bin/ntools_classification_superviewer.cgi) [37]. MapMan was used as the classification source to assign functional categories for each gene [38]. For GO term enrichment analysis, all genes with *P*-value <0.01 and fold change >2.0 were loaded in “Term enrichment” using AmiGO software (http://amigo.geneontology.org) [39]. Normalized frequency (NF) of each functional category was calculated as following: NF  =  sample frequency of each category in this experiment/background frequency of each category in the Arabidopsis genome.

### Cluster analysis

The data sets of specific genes were imported into the CLUSTER program [40], http://bonsai.ims.u-tokyo.ac.jp/~mdehoon/software/cluster/). Hierarchical cluster analysis was performed using an uncentered matrix and complete linkage method. Resulting tree figures were displayed using the software package, Java Treeview (http://jtreeview.sourceforge.net/).

## Results

### Effects of salt stress on seed germination of three ecotypes

**Figure 1:**
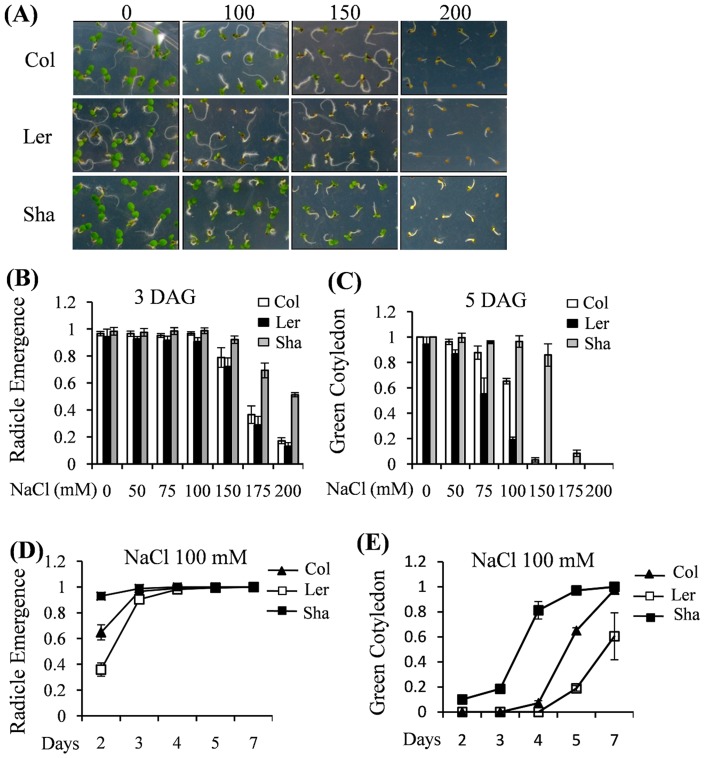
Comparison of seed germination rates among Col, Ler and Sha under salt stress treatment. (A) Plants were grown on MS plate supplied with the indicated concentrations of NaCl (mM). Photos were taken after 5 DAG (Days-After-Germination). Bar = 1 cm. (B) & (C) Germination rates were compared with various concentrations of NaCl. Germination rates were analyzed by counting the number of emergenced radicles after 3 DAG on the indicated concentrations of NaCl in (B) or by counting the number of green cotyledons after 5 DAG in (C). The values indicated means + SEs of four independent experimental repeats (n = 30). (D) & (E) The kinetics of germination time among Col, Ler and Sha were analyzed with the same concentration of NaCl (100 mM). Germination rates were determined by counting the number of emergenced radicles (D) and green cotyledons (E) at the indicated time points. The values indicated means + SEs of four independent experimental repeats (n = 30).

On the fifth Day After Germination (DAG), the Sha ecotype exhibited less injury than Col and Ler in the presence of 150 mM NaCl (Figure 1A). More than half of the Sha seeds (51.3%) developed a radicle even in the presence of 200 mM NaCl at 3 DAG and 86% of the seeds developed green cotyledons at 5 DAG in the presence of 150 mM NaCl, while less than 13% and 3% of other two ecotypes (Col and Ler) seeds developed radicle and green cotyledons, respectively (Figure 1B and C). In the presence of 100 mM NaCl, 93% and 97% of Sha seeds developed radicles at 2 DAG and green cotyledons at 5 DAG, respectively. In comparison, only 36% and 20% of Ler seeds showed radicles and green cotyledons at this stage. Col ecotype showed moderate susceptibility to salt stress and the germination parameters were in between Sha and Ler (Figure 1D and E). Sha ecotype also had relatively longer primary roots and significantly more lateral roots than those of Ler and Col (Figure S1).

### Effects of salt stress on plant growth of three ecotypes

**Figure 2:**
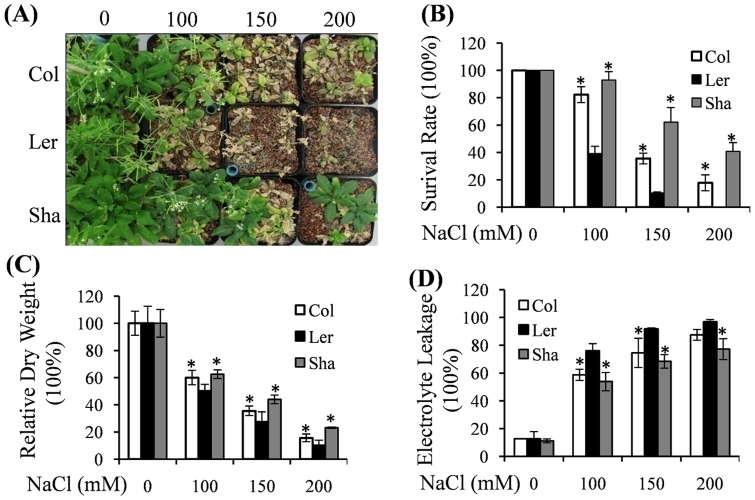
Adult phenotypes analysis among Col, Ler and Sha after salt stress treatment. **Salt treatments were initiated at 14 DAS (Day-After-Sowing).** (A) 2-week-old seedlings of Col, Ler and Sha were treated with indicated concentrations of NaCl for 14 days. Plants were photographed after 2 weeks treatments. (B) Survival rates were calculated from the results of above three independent experiments (n = 20). The values indicated means + SEs. * indicated significant difference with *P*<0.05 (*t-test*) in relative to Ler. (C) Relative dry weight comparison after salt treatment. The values indicated means + SEs of four independent experimental repeats (n = 30). * indicated significant difference with *P*<0.05 (*t-test*) in relative to Ler. (D) Plants were grown for 2 weeks under normal condition and exposed to different concentrations of NaCl treatments. At 10 days after treatment, aerial plants were harvested for measurement of relative electrolyte leakage. The values indicated means + SEs of four independent experimental repeats (n = 15). * indicated significant difference with *P*<0.05 (*t-test*) in relative to Ler.

The survival rate of the Sha ecotype at various concentrations of NaCl was significantly greater relative to the Ler and Col ecotypes (Figure 2A). In the presence of 100 mM NaCl, growth of Sha was less inhibited and 93% of the plants were alive at 14 days after treatment in soil. After 200 mM NaCl treatment, 40% of the Sha plants kept growing while all of the Ler plants died (Figure 2B), resulting in significantly higher dry weight for the Sha ecotype (Figure 2C). The electrolyte leakage test showed that Ler exhibited significantly higher electrolyte leakage than Sha after 10 days salt treatment, indicating Ler sustained less cell membrane stability when compared to Sha (Figure 2D). Col ecotype showed moderate susceptibility to long term salt treatment (Figure 2A–D). Taken together, the enhanced salt tolerance of Sha can be attributed at least in part to increased cell membrane integrity and stability.

### Redox response after salt treatment

**Figure 3:**
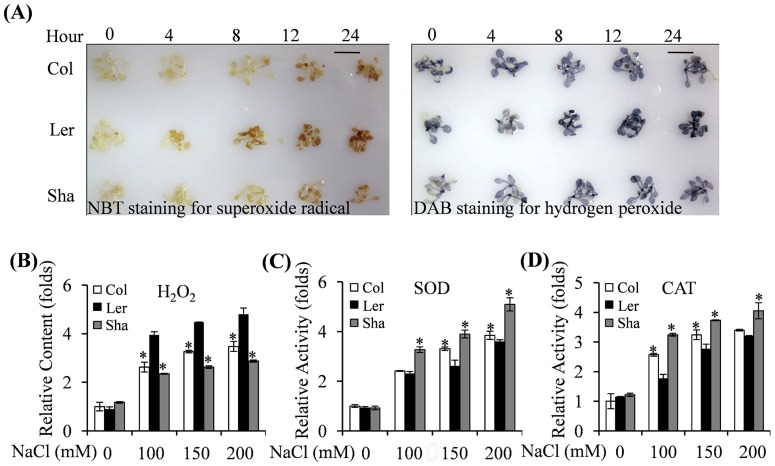
Quantitative comparison of superoxide contents and antioxidant enzyme activities (SOD and CAT) among Col, Ler and Sha after salt stress treatment. Two-week-old plants were started to be treated with the indicated concentrations of NaCl for 10 days before measurement in (B), (C) & (D). The values indicated means + SEs of two independent experimental repeats in (B), (C) & (D) (n = 15). * indicated significant difference with *P*<0.05 (t-test) in relative to Ler. (A) Visualization of superoxide radical and hydrogen peroxide detected by NBT and DAB staining. Detections have been done on 2-week-old MS-grown plants subjected to subsequent treatment with 200 mM NaCl for the indicated time. Bar = 1 cm. (B) Changes in H_2_O_2_ content were analyzed with different salt treatment. (C) Changes in SOD activity were analyzed with different salt treatment. (D) Changes in CAT activity were analyzed with different salt treatment.

After 200 mM NaCl treatment, both superoxide radical and hydrogen peroxide accumulated to a relatively higher content in Ler than in Sha (Figure 3A). A quantification assay indicated that hydrogen peroxide levels in Ler were about 2 folds higher than those in Sha under the various salt conditions (Figure 3B). The SOD and CAT activities in Sha were significantly greater than in Ler after 10 days after salt treatments (Figure 3C and D). ROS content and antioxidant enzyme activities in Col ecotype were in between those of Sha and Ler ecotypes.

### General transcriptomic responses by salinity effect and ecotype effect

**Figure 4:**
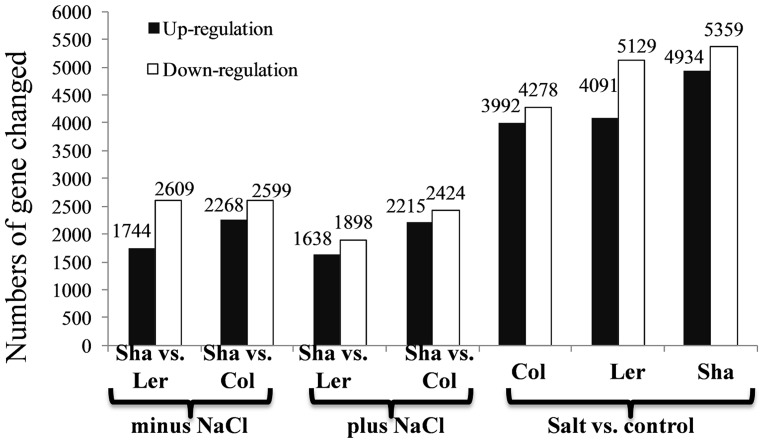
Numbers of gene changed by Sha ecotype effect and salt effect.

In total, the expression levels of 7209 genes were significantly changed by either salt stress (referred to salt effect) or between Sha and other two ecotypes (referred to Sha ecotype effect) (Table S1). In the absence of salt, 4353 and 4867 genes showed differential expression levels in the comparisons of Sha vs. Ler and Sha vs. Col, respectively, while in the presence of salt, the differences between Sha vs. Ler and Col were 3536 and 4639, respectively (Figure 4).

As reported by many groups, *dehydration responsive element binding* (*DREB*)*/C-repeat binding factors* (*CBF*), *LOW-TEMPERATURE-INDUCED* (*LTI*) genes, *DROUGHT-INDUCED* (*DI*) genes, *COLD-REGULATED* (*COR*) genes, *EARLY RESPONSIVE TO DEHYDRATION* (*ERD*) genes, *LATE EMBRYOGEENESIS ABUNDANT* (*LEA*) genes and *KIN1* gene were highly induced by abiotic stress treatments [8], [41], [42]. In this study, expression levels of 20 *DREB/CBF, LTI, DI, CO*R, *ERD, KIN* genes were significantly up-regulated after salt treatment (Figure S2). Moreover, ABA receptor genes (*PYR/PYL*s) were generally down-regulated after salt stress treatment, while other genes involved in ABA pathways, including *PROTEIN PHOSPHATASE 2C* (*PP2C*), *ABA RESPONSIVE ELEMENT-BINDING FACTOR* (*ABF*), ABA metabolism and ABA catabolism, were mainly up-regulated in the presence of salt (Figure S3). These data agreed fairly well with those of [41] that abiotic stress changed ABA pathway related genes. These results provide support for the validity of the data obtained by microarray analysis.

### Cluster and overlap analyses revealed common and contrasting changes by salinity and among ecotypes

**Figure 5:**
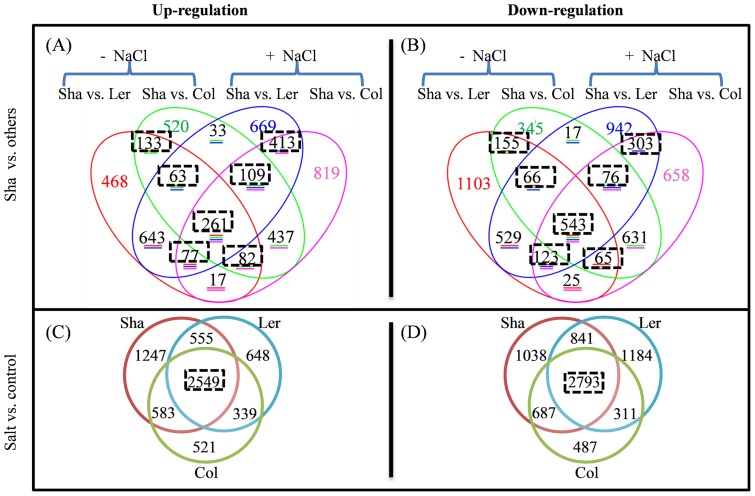
Numbers of overlapping transcripts changed between ecotypes and after salt treatment. Differentially expressed transcripts were those with P<0.05 and fold change >2. Genes commonly regulated between ecotypes or by salt treatment were highlighted in dotted rectangles and the detailed information of these genes was listed as in Table S1.

In total, 1368 and 1905 genes were commonly regulated by Sha vs. Ler and Sha vs. Col in the absence and presence of salt, respectively (Figure 5A and B). Cluster analysis revealed that 52–65% of salt stress affected genes was in common in three ecotypes (Figure S4). Among them, 2549 and 2793 differentially expressed genes were co-regulated by salt in all three ecotypes (Figure 5C and D), indicating common changes after salt stress treatment.

**Figure 6:**
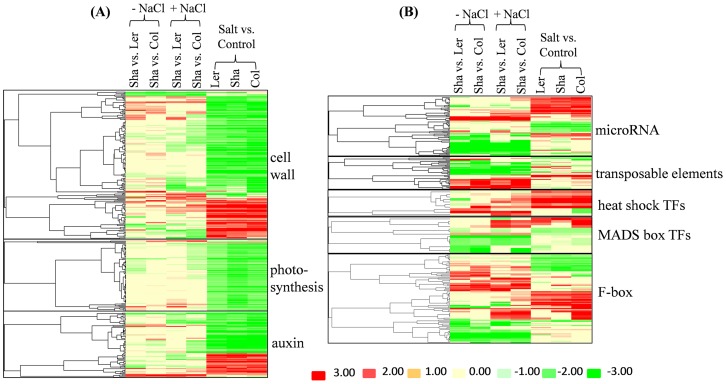
Cluster analyses of transcripts involved in specific pathway. Red, increase in transcript abundance (up-regulation); green, decrease in transcript abundance (down-regulation); yellow, no change. The color scales were also indicated. Hierarchical cluster analysis was applied for differentially expressed transcripts (P < 0.05 and log 2 fold change > 1 or < −1) with Cluster 3.0 software. The resulting tree figures were displayed using the software package, Java TreeView.

Many genes associated with cell walls, photosynthesis (PS), auxin (Figure 6A), secondary metabolism, and biotic stress (Figure S5) were significantly down-regulated after salt treatment, but there were no significant changes between Sha and the other two ecotypes (Figure 6A). In addition, salt treatment also extensively up-regulated genes involved in ABA pathway, carbohydrate metabolism (CHO), ethylene and transport for all three ecotypes (Figure S5). In contrast, expression levels of transposable element genes (TE), microRNA and antisense sequence were significantly changed between Sha and other two ecotypes, and only slightly by salinity (Figure 6B; Table S1). F-box genes, heat shock transcription factors and MADS box transcription factors were also extensively changed between Sha and other two ecotypes (Figure 6B).

### Pathway enrichment analyses of genes changed by Sha ecotype effect and salt effect

Several metabolic pathways such as hormone metabolism, secondary metabolism, TCA, development, and stress (Table 1, Group 1), and GO terms involved in defense and stress responses (Table 2, Group 1) were enriched by both salt effect and Sha ecotype effect, indicating the preconditioned stress tolerance in Sha ecotype. Salt stress treatment extensively changed expression levels of many other genes, resulting in enrichment of related pathways (fermentation, glyoxylate cycle, polyamine metabolism, metal handling, etc) (Table 1, Group 4) and GO terms (regulation of transport and response to most other abiotic stresses) (Table 2, Group 2-3). Pathways involving in microRNA and natural antisense (Table 1, Group 3) were over-represented between the comparison of Sha and other two ecotypes.

**Table 1:** Pathway enrichment analysis showed several pathways were enriched by salinity and ecotype effects.

Groups	MapMan Pathways			Sha vs. Ler/Col		Sha vs. Ler/Col	
		Salt vs. Control		– NaCl		+ NaCl	
		NF^1^	P-value	NF^1^	P-value	NF^1^	P-value
1	hormone metabolism	2.51	0.0000	1.29	0.0320	1.50	0.0020
	secondary metabolism	2.11	0.0000	1.74	0.0012	2.16	0.0000
	Misc	1.87	0.0000	1.91	0.0000	1.64	0.0000
	transport	1.72	0.0000	1.10	0.0480	1.14	0.0270
	development	1.57	0.0000	1.39	0.0063	1.30	0.0082
	TCA/org. transformation	1.40	0.0380	3.21	0.0008	3.04	0.0002
	Stress	1.36	0.0000	2.22	0.0000	2.28	0.0000
2	metal handling	2.00	0.0001	–	–	2.52	0.0018
	nucleotide metabolism	0.73	0.0270	–	–	1.66	0.0160
3	not assigned	0.87	0.0000	1.04	0.0098	1.01	0.0190
	micro RNA, natural antisense etc	0.56	0.0000	1.70	0.0023	1.24	0.0410
	signalling	1.32	0.0000	1.12	0.0340	0.97	0.0480
	RNA	1.05	0.0072	0.78	0.0015	0.85	0.0048
4	fermentation	4.71	0.0000	–	–	–	–
	gluconeogenese/glyoxylate cycle	4.05	0.0002	–	–	–	–
	polyamine metabolism	3.70	0.0002	–	–	–	–
	Biodegradation of Xenobiotics	2.82	0.0003	–	–	–	–
	C1-metabolism	1.97	0.0082	–	–	–	–
	amino acid metabolism	1.81	0.0000	–	–	–	–
	minor CHO metabolism	1.70	0.0007	–	–	–	–
	tetrapyrrole synthesis	1.51	0.0500	–	–	–	–
	lipid metabolism	1.27	0.0038	–	–	–	–
	PS	2.68	0.0000	0.12	0.0025	0.27	0.0034
	major CHO metabolism	2.13	0.0000	–	–	0.00	0.0037
	cell wall	1.89	0.0000	0.62	0.0180	–	–
	cell	0.96	0.0350	–	–	–	–
	mitochondrial electron transport/ATP synthesis	0.58	0.0072	–	–	–	–
	Co-factor and vitamine metabolism	0.24	0.0008	–	–	–	–
5	protein	0.58	0.0000	0.64	0.0000	0.69	0.0000
	DNA	0.17	0.0000	0.40	0.0000	0.32	0.0000

1 NF: Normalized frequency  =  sample frequency of each category in this experiment/background frequency of each category in the Arabidopsis genome.

Genes with P-value <0.05 and fold change >2.0 were loaded and annotated in the Classification SuperViewer Tool (http://bar.utoronto.ca/ntools/cgi-bin/ntools_classification_superviewer.cgi). MapMan was used as the classification source to assign functional categories for each gene. Group 1: pathways enriched by both salt and ecotype effects; Group 2: pathways enriched by salt effect and ecotype effect in the presence of salt; Group 3: pathways mainly enriched by salt effect; –: no significant enrichment. The color scales indicated different normalized frequencies which were described in Materials and Methods.

**Table 2:** Stress-related GO term enrichment analysis.

Groups	GO Terms	Salt vs. Control		minus salt		plus salt	
				Sha vs. other ecotypes		Sha vs. other ecotypes	
		P-value	NF^1^	P-value	NF^1^	P-value	NF^1^
1	GO:0006950response to stress	0.0000	1.59	0.0000	1.45	0.0236	1.24
	GO:0006952defense response	0.0000	1.57	0.0000	1.75	0.0181	1.48
	GO:0050896response to stimulus	0.0000	1.52	0.0040	1.26	–	–
	GO:0007154cell communication	0.0000	1.67	0.0330	1.46	–	–
2	GO:0044277cell wall disassembly	0.0105	5.01	–	–	–	–
	GO:0006812cation transport	0.0000	2.05	–	–	–	–
	GO:0055080cation homeostasis	0.0008	1.98	–	–	–	–
	GO:0050801ion homeostasis	0.0006	1.92	–	–	–	–
	GO:0051049regulation of transport	0.0007	2.10	–	–	–	–
	GO:0043269regulation of ion transport	0.0002	2.24	–	–	–	–
	GO:0006820anion transport	0.0000	2.49	–	–	–	–
	GO:0010155regulation of proton transport	0.0139	2.54	–	–	–	–
3	GO:0009628response to abiotic stimulus	0.0000	1.51	–	–	–	–
	GO:0009607response to biotic stimulus	0.0000	1.68	–	–	–	–
	GO:0009737response to abscisic acid stimulus	0.0000	2.31	–	–	–	–
	GO:0009269response to desiccation	0.0131	3.28	–	–	–	–
	GO:0009414response to water deprivation	0.0000	3.05	–	–	–	–
	GO:0009408response to heat	0.0000	2.17	–	–	–	–
	GO:0006979response to oxidative stress	0.0000	1.79	–	–	–	–
	GO:0009651response to salt stress	0.0000	1.72	–	–	–	–
	GO:0006970response to osmotic stress	0.0000	1.72	–	–	–	–
	GO:0009409response to cold	0.0000	1.72	–	–	–	–

Term enrichment analysis was performed using AmiGO software.

1 NF: Normalized frequency  =  sample frequency of each category in this experiment/background frequency of each category in the Arabidopsis genome.

### Specific genes regulated by Sha ecotype effect and salt effect

In total, 98 of the 218 genes which were differentially expressed by Sha relative to both Ler and Col also showed significantly expression changes after salt treatment, including 29 up-regulated genes and 69 down-regulated genes (Table 3). This set of genes mainly functions in secondary metabolism, hormone metabolism, regulation of transcription, protein metabolism, and signaling (Table 3A, B). There were several genes with increased or decreased expression in Sha relative to Ler and Col exhibited the opposite trend in response to salt (Table 3C and D). For example, one extensin gene (AT1G26250), one oxidase gene (AT5G23980), and one methyltransferase (AT1G77530) were down-regulated under salt stress condition, but exhibited 2 to 16-fold increases in transcript abundance in Sha in relative to the other two ecotypes (Table 3C). Meanwhile, several genes with annotated function in secondary metabolism, biotic stress, and transcriptional regulation were up-regulated by salt stress, but down-regulated in Sha. It’s worth mentioning that one oxidoreductase gene (AT5G54190) and one transposase gene (AT3G02515) were up-regulated by salt 2 to 4-fold, but showed up to 1038-fold decreases in Sha in comparison to Ler and Col (Table 3D).

**Table 3:** List of genes commonly regulated by salt treatment and in Sha ecotype.

locus	– NaCl		+ NaCl		Salt vs. Control			MapMan BIN	GeneName
	Sha vs. Ler	Sha vs. Col	Sha vs. Ler	Sha vs. Col	Col	Ler	Sha		
**A: Up-regulation by Sha effect and salt effect**									
AT2G02560	2.33	1.95	2.17	2.19	0.97	1.37	1.21	[17.2.2] hormone metabolism	CAND1
AT5G43570	1.89	2.44	2.38	2.85	2.25	2.19	2.67	[20.1.7.6] stress.biotic	PR protein
AT5G57260	2.16	2.68	2.67	3.57	0.81	1.19	1.70	[26.10] misc.cytochrome P450	CYP71B10
AT1G73010	1.14	1.31	1.00	1.28	3.49	3.60	3.45	[26.13] misc.acid and phosphatases	phosphatase
AT4G24890	3.99	3.70	3.52	3.75	1.02	1.54	1.07	[26.13] misc.acid and phosphatases	acid phosphatase
AT5G62080	1.96	2.31	1.11	1.50	2.26	2.31	1.46	[26.21] misc.protease inhibitor	protease inhibitor
AT2G46880	4.25	1.68	1.33	1.94	1.60	4.79	1.86	[26.27] misc.phosphoesterase	PAP14
AT1G59670	2.34	2.93	3.36	3.04	3.33	2.43	3.45	[26.9] misc.glutathione S transferases	ATGSTU15
AT4G16160	1.99	2.52	1.00	2.14	3.74	4.34	3.35	[29.3.2] protein.targeting.mitochondria	ATOEP16-2
AT1G53080	1.73	1.87	1.29	1.20	5.91	5.69	5.25	[29.4] protein.postranslational mod.	lectin
AT3G10510	2.89	1.71	2.19	1.55	3.21	3.74	3.05	[29.5.11.4.3.2] protein.degradation	F-box
AT1G67000	1.50	2.72	1.66	1.90	1.95	0.99	1.14	[30.2.20] signalling.receptor kinases	protein kinase
AT3G18610	1.12	1.42	1.17	2.28	1.55	2.36	2.40	[30.5] signalling.G-proteins	nucleolin
AT2G25340	1.56	2.64	2.01	3.14	2.83	2.88	3.32	[31.4] cell.vesicle transport	ATVAMP712
AT2G41260	5.55	2.70	1.92	4.02	−0.45	4.50	0.87	[33.99] development.unspecified	LEA
AT1G04560	2.31	2.55	2.31	2.67	6.06	6.17	6.17	[34.99] transport.misc	AWPM-19-like
AT5G55070	1.30	1.22	1.42	2.11	0.81	1.57	1.69	[8.1.5] TCA/org. transformation.TCA	dehydrogenase
**B: Down-regulation by Sha effect and salt effect**									
AT2G15050	−3.34	−3.97	−3.63	−4.07	−1.13	−0.94	−1.23	[11.6] lipid metabolism	LTP; lipid binding
AT4G15340	−1.01	−3.37	−2.40	−3.05	−2.60	−0.88	−2.27	[16.1.5.1003] secondary metabolism	ATPEN1
AT1G34060	−2.50	−1.45	−1.25	−2.38	−0.44	−2.63	−1.38	[16.5.99.1] secondary metabolism	alliinase
AT1G34490	−3.10	−1.32	−3.44	−2.65	−2.98	−3.97	−4.32	[16.7.1001] secondary metabolism	wax synthase
AT5G18020	−2.51	−2.71	−1.71	−2.83	−2.69	−3.61	−2.81	[17.2.3] hormone metabolism.auxin	auxin-responsive
AT1G56680	−1.41	−1.21	−2.34	−2.60	−2.91	−3.37	−4.30	[20.1.1001] stress.biotic	glycoside hydrolase
AT2G15080	−4.48	−3.89	−3.83	−3.51	−3.00	−3.28	−2.62	[20.1.7] stress.biotic.PR-proteins	AtRLP19
AT4G19530	−1.38	−1.81	−1.76	−2.48	−1.45	−1.75	−2.13	[20.1.7] stress.biotic.PR-proteins	disease resistance
AT3G46940	−1.19	−1.07	−2.13	−1.96	−1.20	−1.14	−2.09	[23.5.5] nucleotide metabolism	nucleotidohydrolase
AT5G03350	−3.95	−2.65	−5.67	−4.46	−2.21	−2.30	−4.02	[26.16] misc.myrosinases	lectin
AT1G54000	−1.15	−1.12	−1.75	−1.40	−1.27	−0.96	−1.56	[26.16] misc.myrosinases	myrosinase
AT3G43670	−1.47	−1.25	−1.97	−2.75	−0.35	−1.35	−1.85	[26.7] misc.oxidases − copper, flavone	amine oxidase
AT1G63100	−1.73	−1.46	−1.59	−1.48	−1.46	−1.62	−1.48	[27.3.21] RNA.transcription regulation	scarecrow TF
AT3G27920	−1.06	−2.00	−1.82	−2.00	−1.75	−0.99	−1.75	[27.3.25] RNA.transcription regulation	MYB
AT1G26680	−1.64	−2.24	−1.63	−2.36	−1.06	−1.19	−1.19	[27.3.41] RNA.transcription regulation	B3 TF
AT5G10570	−2.39	−1.21	−2.09	−1.38	−1.86	−2.34	−2.03	[27.3.6] RNA.transcription regulation	Bhlh
AT5G44560	−1.06	−1.63	−1.84	−1.70	−1.05	−0.33	−1.12	[27.3.71] RNA.transcription regulation	VPS2.2
AT1G03420	−1.36	−2.60	−2.13	−3.44	−0.93	−1.00	−1.77	[28.1.1] DNA.transposase	oxidoreductase
AT5G44635	−1.19	−1.85	−1.50	−2.15	−1.29	−1.28	−1.60	[28.1] DNA.synthesis	MCM protein
AT1G56720	−1.16	−1.53	−1.14	−1.84	−0.84	−1.17	−1.15	[29.4.1.55] protein.kinase	protein kinase
AT3G07070	−1.66	−1.53	−3.23	−2.78	−2.57	−2.24	−3.82	[29.4.1.57] protein.kinase	protein kinase
AT1G48260	−3.59	−4.02	−4.27	−3.56	−1.55	−0.40	−1.09	[29.4] protein.postranslat. modification	CIPK17
AT4G20430	−1.53	−1.65	−1.69	−2.22	−0.93	−1.34	−1.50	[29.5.1] protein.degradation.subtilases	subtilase
AT3G44120	−1.73	−1.43	−1.15	−1.82	−1.07	−2.03	−1.45	[29.5.11] protein.ubiquitin.E3	F-box
AT3G51350	−1.89	−1.82	−2.53	−2.60	−3.01	−3.16	−3.79	[29.5.4] protein.degradation	aspartyl protease
AT4G39710	−3.05	−2.98	−3.15	−3.63	−3.51	−4.06	−4.16	[29.6] protein.folding	immunophilin
AT3G50840	−1.95	−1.64	−2.20	−2.67	−1.38	−2.17	−2.42	[30.11] signalling.light	NPH3protein
AT5G39030	−3.93	−3.09	−3.67	−2.61	−1.32	−1.09	−0.83	[30.2.16] signalling.receptor kinases	protein kinase
AT1G11280	−4.18	−4.19	−4.34	−4.74	−0.73	−1.12	−1.28	[30.2.24] signalling.receptor kinases	protein kinase
AT5G49760	−1.64	−1.28	−1.58	−1.22	−1.04	−1.04	−0.98	[30.2.8.1] signalling.receptor kinases	protein kinase
AT1G51805	−1.75	−2.30	−1.13	−2.67	−1.26	−2.24	−1.62	[30.2.99] signalling.receptor kinases	protein kinase
AT3G47220	−1.03	−1.35	−3.14	−3.20	−1.02	−0.76	−2.87	[30.4.4] signalling.phosphinositides	phospholipase C
AT5G02370	−1.28	−1.36	−1.91	−1.75	−1.61	−1.36	−2.00	[31.1] cell.organisation	kinesin
AT1G73690	−1.16	−1.25	−2.42	−1.92	−1.01	−0.42	−1.68	[31.2] cell.division	CDKD1;1
AT3G62030	−1.64	−1.52	−1.14	−1.24	−1.03	−1.25	−0.75	[31.3.1] cell.cycle. isomerase	ROC4
AT2G26760	−1.28	−1.46	−1.73	−1.72	−1.52	−1.33	−1.77	[31.3] cell.cycle	CYCB1;4
AT3G05480	−1.51	−2.34	−1.80	−2.51	−1.18	−1.06	−1.35	[31.3] cell.cycle	RAD9
AT1G66725	−2.70	−1.81	−2.04	−1.95	−0.80	−1.60	-0.93	[32] micro RNA, natural antisense etc	miscRNA
AT4G22233	−1.87	−2.39	−1.27	−2.91	−0.65	−1.76	−1.16	[32] micro RNA, natural antisense etc	miscRNA
AT2G21045	−3.05	−2.63	−2.95	−2.33	−1.66	−1.46	−1.36	[33.99] development.unspecified	hypothetical protein
**C: Up-regulation by Sha effect and dow-regulation by salt effect**									
AT1G26250	1.70	1.98	1.02	1.31	−3.10	−3.10	−3.78	[10.5.3] cell wall proteins.LRR	extensin
AT5G23980	4.34	1.18	3.87	2.32	−4.44	−2.82	−3.30	[15.1] metal handling.acquisition	oxidase
AT1G77530	1.59	1.70	1.33	1.01	−3.70	−4.14	−4.40	[16.2] secondary metabolism	methyltransferase
AT5G63595	1.92	2.13	1.43	1.75	−1.67	−1.55	−2.04	[16.8.4] secondary metabolism	flavonol synthase
AT5G38020	2.66	1.84	2.64	1.38	−0.90	−1.34	−1.36	[17.8] hormone.salicylic acid	methyltransferase
AT2G21550	2.99	1.57	3.67	1.97	−1.47	−1.76	−1.07	[25] C1-metabolism	DHFR-TS
AT5G38540	5.52	3.08	4.91	3.73	−3.81	−2.55	−3.16	[26.16] misc.myrosinases	jacalin lectin
AT2G19910	3.03	1.56	2.66	1.36	−1.47	−1.30	−1.67	[27.2] RNA.transcription	RNA polymerase
AT2G19410	1.18	1.03	1.17	1.03	−2.43	−2.42	−2.43	[29.4.1] protein.kinase	protein kinase
AT2G03200	1.34	1.08	1.42	1.03	−1.58	−1.71	−1.63	[29.5.4] protein.degradation	aspartyl protease
AT5G59670	1.17	1.82	1.96	1.35	−1.97	−3.24	−2.44	[30.2.1] signalling.receptor kinase	protein kinase
AT3G45680	1.62	1.54	2.12	1.44	−1.06	−1.67	−1.17	[34.13] transport.peptides	transport
**D: Down-regulation by Sha effect and up-regulation by salt effect**									
AT4G08870	−2.30	−2.40	−2.44	−2.29	1.87	2.12	1.98	[13.2] amino acid.degradation	arginase
AT1G61120	−4.23	−1.45	−5.14	−2.52	5.12	4.96	4.05	[16.1] secondary metabolism	terpene synthase
AT1G52040	−1.49	−1.84	−1.88	−2.50	3.32	3.04	2.66	[16.5] secondary metabolism	MBP1
AT4G22870	−1.16	−2.38	−1.13	−1.82	2.09	2.62	2.65	[16.8] secondary metabolism	dioxygenase
AT5G42800	−2.48	−2.46	−2.09	−1.99	2.69	2.77	3.16	[16.8] secondary metabolism	DFR
AT5G54190	−7.06	−4.45	−7.09	−3.53	1.48	2.43	2.41	[19.14] tetrapyrrole synthesis	oxidoreductase
AT1G45616	−4.96	−6.17	−3.36	−4.93	3.99	3.63	5.24	[20.1.1001] stress.biotic	AtRLP6
AT3G44670	−3.06	−4.43	−1.46	−3.31	1.10	0.61	2.22	[20.1.2] stress.biotic.receptors	receptor
AT3G59930	−2.43	−3.40	−1.45	−1.20	1.76	2.98	3.97	[20.1.7] stress.biotic.PR-proteins	hypothetical protein
AT5G33355	−2.67	−3.45	−1.33	−1.16	1.81	2.77	4.10	[20.1.7] stress.biotic.PR-proteins	hypothetical protein
AT4G15910	−3.06	−2.57	−1.79	−1.63	2.60	2.27	3.54	[20.2.3] stress.abiotic.drought/salt	ATDI21
AT4G14090	−1.25	−2.04	−1.34	−2.02	2.47	2.58	2.49	[26.2] misc.UDPG transferases	glucosyl transferase
AT2G39030	−3.24	−2.00	−1.25	−1.04	8.02	6.99	8.98	[26.24] misc.N-acetyltransferase	acetyltransferase
AT2G43660	−1.66	−3.01	−1.25	−1.42	2.27	3.46	3.87	[26.4] misc.beta 1,3 glucan hydrolases	glycosyl hydrolase
AT1G62580	−4.03	−3.20	−4.91	−5.71	4.63	3.00	2.12	[26.7] misc.oxidases – copper, flavone	monooxygenase
AT2G23620	−2.85	−1.17	−2.75	−1.34	1.21	0.95	1.04	[26.8] misc.nitrilases, *nitrile lyase	hydrolase
AT3G01540	−1.48	−1.61	−1.77	−1.49	0.89	1.31	1.02	[27.1.2] RNA.RNA helicase	DRH1
AT4G12350	−3.56	−3.63	−3.41	−2.55	1.45	2.38	2.53	[27.3] RNA.regulation of transcription	MYB42
AT5G46830	−2.66	−3.86	−1.60	−2.61	2.12	2.31	3.37	[27.3] RNA.regulation of transcription	bHLH
AT4G20970	−1.75	−2.13	−1.04	−1.55	3.25	3.14	3.84	[27.3] RNA.regulation of transcription	bHLH
AT1G51700	−1.22	−1.33	−1.21	−1.24	1.59	1.68	1.69	[27.3] RNA.regulation of transcription	ADOF1
AT3G02515	###	−5.22	−8.26	−4.02	1.00	0.44	2.20	[28.1] DNA.transposase	transposase
AT5G43580	−3.89	−2.56	−2.52	−1.77	1.74	1.16	2.53	[29.5] protein.degradation	peptidase inhibitor
AT1G66830	−1.60	−1.26	−1.96	−1.38	2.79	3.04	2.68	[30.2.3] signalling.receptor kinases	protein kinase
AT1G53160	−1.14	−1.89	−2.32	−2.27	4.47	5.27	4.09	[33.3] development.SPL	SPL4
AT3G54150	−2.74	−1.48	−2.29	−1.02	1.74	1.75	2.20	[33.99] development.unspecified	embryo-abundant
AT3G49620	−1.20	−1.10	−4.38	−1.36	2.02	4.94	1.76	[33.99] development.unspecified	DIN11
AT4G18210	−2.28	−1.65	−2.63	−2.21	1.60	1.39	1.05	[34.10] transport.nucleotides	ATPUP10
AT2G04070	−4.29	−2.59	−6.15	−4.36	3.69	3.78	1.92	[34.99] transport.misc	antiporter

Black background means an increase (denoted by up-regulation in the heading) in transcript abundance, and grey background means a decrease (denoted by the heading down-regulation) in transcript abundance. Fold changes are log 2 values.

## Discussion

In this work, natural variations in salt tolerance among Sha, Ler and Col ecotypes were analyzed based on their responses to salt treatment in seed germination, root growth, and performance of adult plants in soil (Figure 1–3; Figure S1). All of these results were consistent with previous studies where Ler and Col were observed to be relatively salt-sensitive, but Sha was salt-tolerant [14], [15], [29].

Abiotic stresses cause oxidative stress via rapid and excessive production of reactive oxygen species (ROS), which can lead to oxidative damages [43], [44]. To scavenge the over-production of ROS, plants have developed complex antioxidant defense systems, including antioxidant enzymes like SOD, CAT, and POD. Physiological analyses in this study indicated that salt tolerant Sha ecotype exhibited higher antioxidant enzyme activities and thus less accumulation of ROS than those of other two ecotypes (Figure 3). EL, as one indicator of cell membrane stability, has been widely used to evaluate the extent of cell injury when subjected to various environmental stresses [26]. As the most salt susceptible ecotype, Ler showed highest EL when compared to that in Sha and Col after salt treatment (Figure 2D), indicating that Ler suffered the most severe cell injury. This result was consistent with highest accumulation of ROS and lowest survival in Ler ecotype (Figure 2B).

To date, transcriptional level changes involved in salt tolerant Sha ecotypes were still largely unknown.Our study differs from prior transcriptome analyses in two important ways that are worth considering. First, two salt susceptible Arabidopsis ecotypes (Ler and Col) besides salt tolerant Sha ecotype were used in this study to narrow down the gene list for characterization of salt stress responsive genes in the Sha ecotype. Second, Agilent-021169 Arabidopsis 4 Oligo Microarray were used because many genes were missed in the widely used Affymetrix ATH1 array (like ABA receptors *PYL10-13*).

The transcriptome data here revealed that half of salinity affected genes were commonly up-regulated or down-regulated in all three ecotypes (Figure 5C). In a previous report, we also observed that the majority of salt affected genes were in common between Wassilewskija (Ws) and Col ecotypes [41]. These stress inducible genes played either protective (positive) role or damaging (negative) role and led to abiotic stress adaptation in plants. To characterize a single protective or damaging gene is difficult because overexpression of several genes increases plant stress tolerance but inhibits plant growth as well [41], [42].

Pathway enrichment analysis is an effective approach to characterize “gene networks” after treatment. We observed that redox related genes encoding thioredoxin, ascorbate and glutathione and glutaredoxins were mainly repressed by salinity (Table S1), in which caused increased ROS levels in tested ecotypes (Figure 3A). In addition, our results indicated that fermentation, photosynthesis, polyamine metabolism, secondary metabolism, hormone metabolism and stress related pathways were over-represented after salinity treatment (Table 1). Many genes involved in light reactions, photorespiration and the Calvin cycle were uniformly down-regulated after salt stress treatments (Table S1). Increase of fermentation related genes (encoding aldehyde dehydrogenase, pyruvate decarboxylase-2, and alcohol dehydrogenase) and inhibition of photosynthesis related genes (encoding PSI and PS II polypeptide subunits, Calvin cycle related proteins) (Table S1) by salt stress might be involved in inhibition of plant growth and development.

Further overlap analysis showed that about 30% genes were commonly regulated by both Sha vs. Ler and Sha vs. Col (Figure 5A and B; Figure S4). These results confirmed that extensive transcriptional diversity exists among Arabidopsis ecotypes. Interestingly, expression levels of many transposable element (TE) and microRNA (miRNA) were significantly changed in the comparisons of Sha vs. Ler and Sha vs. Col for up to 1351-fold increases (AT5G27345) and 1629-fold decreases (AT2G13665) (Figure 6B; Table S2). TEs are referred as “controlling elements” in plants [45] and transposon activation in response to abiotic stress has been reported [46], [47]. miRNAs play essential roles in regulating plant stress responses [48]. Therefore, extensive changes of expression of TEs and miRNAs in the comparisons of Sha vs. Ler and Sha vs. Col here indicated that these genes could be involved in salt tolerance of Sha ecotype.

There also were extensive differences in gene expression between Sha and the other two ecotypes for transcription factors (TFs), including heat shock TFs (HSF) and MADS box TFs (Figure 6B). It has been reported that the HSF function as transcriptional activators and directly regulate the expression of various abiotic stress responsive genes [49], [50]. Arabidopsis with overexpression of *AtHSFA2* and transgenic tobacco with sunflower *HSFA9* conferred increased tolerance to severe environmental stresses [50], [51]. Plant MADS-box genes were involved in flowering-time control, reproductive organ development, and vegetative growth [52], [53]. In this study, MADS-box genes were mainly down-regulated when comparing Sha to other two ecotypes, indicating these genes might also function in stress responses. Moreover, several F-box genes were changed in the comparisons of Sha vs. Ler and Sha vs. Col (Figure 6B). In plants, many F-box proteins are targets of microRNAs [54] which showed differential expression between Sha and the other two ecotypes (Figure 6B). One F-box protein, TIR1, is actually an auxin receptor [55], [56]. These changes in the Sha ecotype might contribute to enhanced salt tolerance relative to Col and Ler.

**Figure 7:**
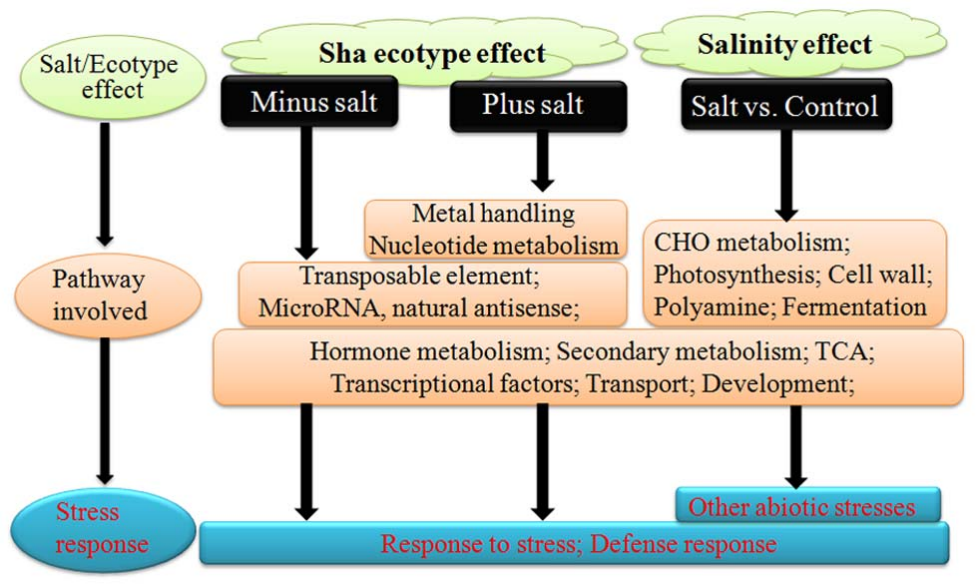
Model of salt effect and ecotype effects.

Transcript levels commonly or contrastingly changed by both salt and the Sha ecotype were of particular interest (Table 3). Further studies are needed to understand the detailed functions of genes that are differentially expressed between Sha and the other two ecotypes. In summary, the Sha ecotype exhibited increased salt tolerance when compared to Ler and Col. One possible model related to salt tolerance of Sha is depicted in Figure 7. Genes involved in CHO metabolism, photosynthesis, cell wall, polyamine and fermentation were extensively changed by salinity effect, while TEs and miRNA related genes were mainly related to the Sha ecotype effect. Other pathways including hormone metabolism, secondary metabolism, TCA, transcriptional factors, transport and development were changed by both salinity and Sha ecotype effects (Figure 7). Therefore, the Sha ecotype showed increased tolerance to stress and defense response, while salt treatment induced tolerance to other abiotic stresses like heat, cold, drought and salt (Table 2). Our results suggest that the Sha ecotype is possibly preconditioned to abiotic stress when compared to Ler and Col through regulation of signaling pathways and stress responsive gene expression. Further studies about the detailed functions of differentially expressed genes between Sha and other two ecotypes are needed.

## Supporting Information

Figure S1 Effect of salt treatment on root growth of Sha, Col and Ler ecotypes.
(TIF)

Figure S2 Expression changes of stress responsive genes by salt effect and Sha ecotype effect.
(TIF)

Figure S3 Cluster analyses of genes involved in ABA signaling transduction pathway.
(TIF)

Figure S4 Cluster analyses of all differentially expressed genes by salt treatment or among ecotypes.
(TIF)

Figure S5 Cluster analyses of specific pathway related genes.
(TIF)

Table S1 Total gene lists differentially expressed by salt treatment or among ecotypes.
(XLSX)

Table S2 Transposable element, miroRNA and histone related transcripts differentially expressed by salt treatment or among ecotypes.
(XLSX)
