# Supplementary figures and images for: Transcriptomic and Physiological Variations of Three Arabidopsis Ecotypes in Response to Salt Stress
Source: PLoS One. 2013 Jul 23;8(7):e69036. doi: 10.1371/journal.pone.0069036 (PMC3720874; doi:10.1371/journal.pone.0069036)

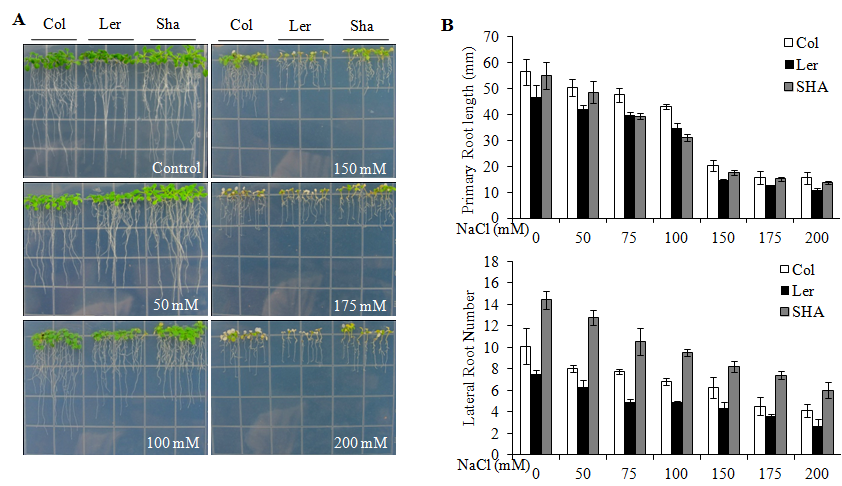

Supplement: Figure S1 — Effect of salt treatment on root growth of Sha, Col and Ler ecotypes. (TIF) [file pone.0069036.s001.tif]

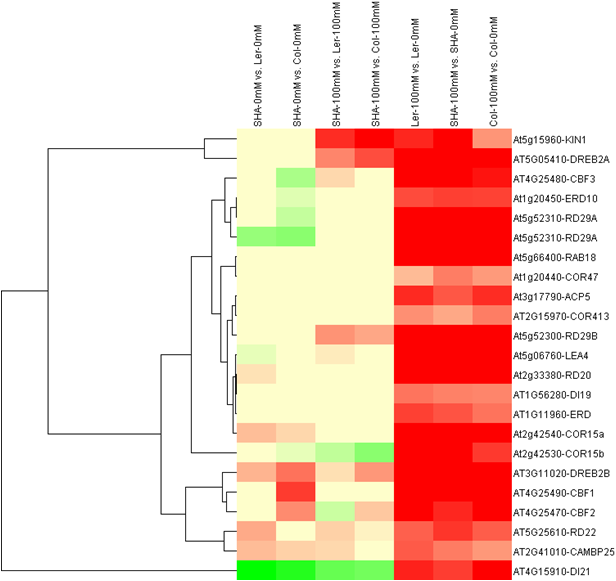

Supplement: Figure S2 — Expression changes of stress responsive genes by salt effect and Sha ecotype effect. (TIF) [file pone.0069036.s002.tif]

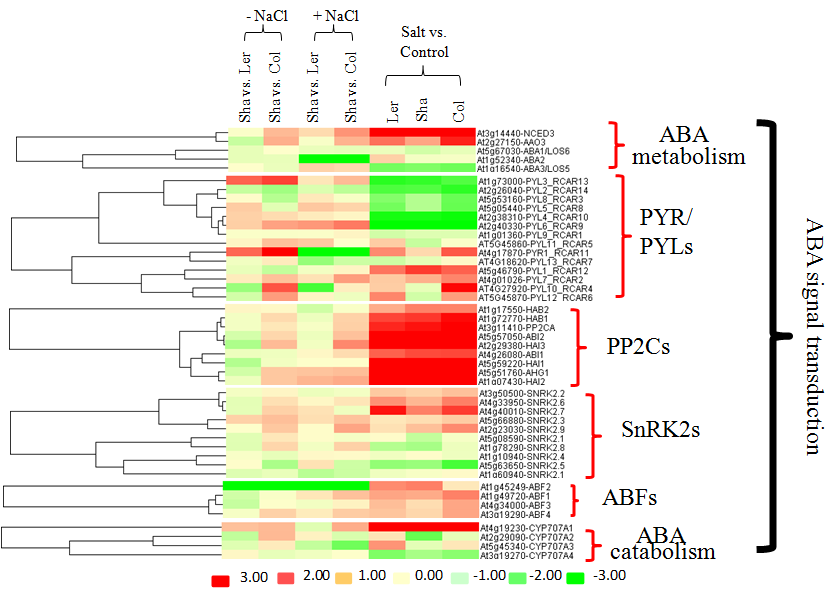

Supplement: Figure S3 — Cluster analyses of genes involved in ABA signaling transduction pathway. (TIF) [file pone.0069036.s003.tif]

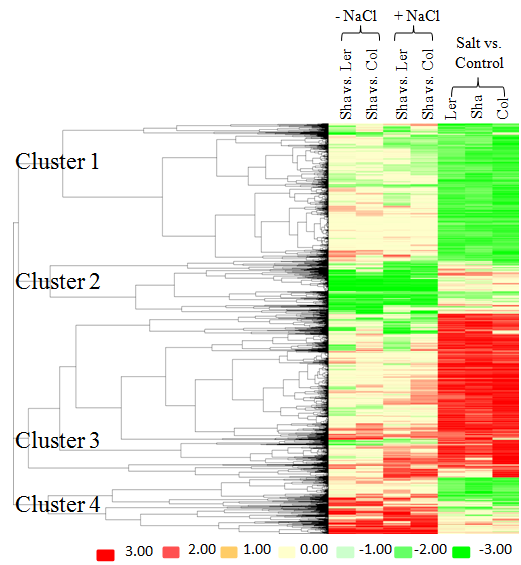

Supplement: Figure S4 — Cluster analyses of all differentially expressed genes by salt treatment or among ecotypes. (TIF) [file pone.0069036.s004.tif]

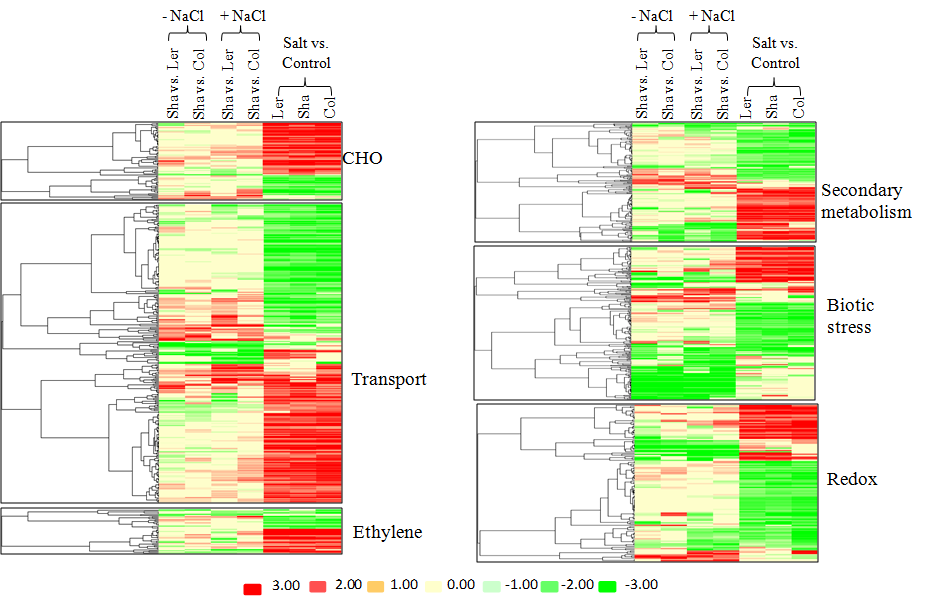

Supplement: Figure S5 — Cluster analyses of specific pathway related genes. (TIF) [file pone.0069036.s005.tif]
